# Chronic Dietary Administration of the Glycolytic Inhibitor 2-Deoxy-D-Glucose (2-DG) Inhibits the Growth of Implanted Ehrlich’s Ascites Tumor in Mice

**DOI:** 10.1371/journal.pone.0132089

**Published:** 2015-07-02

**Authors:** Saurabh Singh, Sanjay Pandey, Anant Narayan Bhatt, Richa Chaudhary, Vikas Bhuria, Namita Kalra, Ravi Soni, Bal Gangadhar Roy, Daman Saluja, Bilikere S. Dwarakanath

**Affiliations:** 1 Division of Radiation Biosciences, Institute of Nuclear Medicine and Allied Sciences, Brig. SK Mazumdar Road, Delhi, India; 2 Medical Biotechnology Laboratory, Dr B.R Ambedkar Center for Biomedical Research, University of Delhi, Delhi, India; Shanghai University of Traditional Chinese Medicine, CHINA

## Abstract

**Background:**

Dietary energy restriction (DER) has been well established as a potent anticancer strategy. Non-adoption of restricted diet for an extended period has limited its practical implementation in humans with a compelling need to develop agents that mimic effects similar to DER, without reduction in actual dietary intake. Glycolytic inhibitor, 2-deoxy-D-glucose (2-DG), has recently been shown to possess potential as an energy restriction mimetic agent (ERMA). In the present study we evaluated the effect of dietary 2-DG administration on a mouse tumor model, with a focus on several potential mechanisms that may account for the inhibition of tumorigenesis.

**Methodology/Principal Findings:**

Swiss albino strain ‘A’ mice were administered with 0.2% and 0.4% w/v 2-DG in drinking water for 3 months prior to tumor implantation (Ehrlich’s ascites carcinoma; EAC) and continued till the termination of the study with no adverse effects on general physiology and animal growth. Dietary 2-DG significantly reduced the tumor incidence, delayed the onset, and compromised the tumor growth along with enhanced survival. We observed reduced blood glucose and serum insulin levels along with decreased proliferating cell nuclear antigen (PCNA) and bromodeoxyuridine positive (BrdU^+^) tumor cells in 2-DG fed mice. Also, reduced levels of certain key players of metabolic pathways such as phosphatidylinositol 3-kinase (PI3K), phosphorylated-Akt and hypoxia inducible factor-1 alpha (HIF-1α) were also noted in tumors of 2-DG fed mice. Further, decrease in CD4^+^/CD8^+^ ratio and T-regulatory cells observed in 2-DG fed mice suggested enhanced antitumor immunity and T cell effector function.

**Conclusion/Significance:**

These results strongly suggest that dietary 2-DG administration in mice, at doses easily achievable in humans, suitably modulates several pleotrophic factors mimicking DER and inhibits tumorigenesis, emphasizing the use of ERMAs as a promising cancer preventive strategy.

## Introduction

Dietary energy restriction (DER) often referred to as ‘under nutrition without malnutrition’ has been extensively shown to reduce the incidence and growth of spontaneous as well as transplanted tumors in rodents [1–3]. This DER, also known as caloric restriction (CR) or energy restriction (ER) is a potent inhibitor of the process of carcinogenesis. It has been hypothesized that DER primarily targets neoplastic cells by reducing net energy status and particularly the glucose metabolism thereby lowering the metabolism of transformed cells, since more than 75% of the tumor types rely on aerobic glycolysis (Warburg’s effect) [4, 5]. Besides its anti-cancer effects, DER has also been shown to delay the aging and reduce the age-associated diseases in variety of model systems including yeast, nematodes, worms, flies, fish, mice, rats, dogs and monkeys [6, 7].

Despite the beneficial effects of DER, it is associated with limitations of essential nutrient deprivation and practical implications of restricted dietary regimens in humans. This has resulted in exploring potential energy restriction mimetic agents (ERMAs), which can provide beneficial effects similar to DER without reducing the actual dietary/calorie intake. In the recent years, several compounds such as resveratrol [8, 9], metformin [10] and 2-deoxy-D-glucose (2-DG) [11, 12] have been shown to possess tumor inhibitory and anti-aging effects indicating their potential to act as ERMAs. Since tumors show high dependency on glycolysis as a result of metabolic reprogramming associated with tumorigenesis, there is a considerable amount of interest in exploring the energy restriction mimetic 2-DG as a potent anti-neoplastic agent.

The precise mechanisms underlying the beneficial effects of DER are not completely understood, although it is well established that DER is associated with decrease in levels of blood glucose, serum insulin and growth factors such as insulin-like growth factor-1 (IGF-1) which are involved in tumorigenesis [6, 13]. Besides direct effects such as anti-proliferative [14], pro-apoptotic [15] on tumors, DER is also known to modulate the immune status [16], anti-oxidant defense system [17], extra cellular matrix (ECM) [2] and tumor associated angiogenesis [18] as a consequence of adaptive response for the metabolic stress posed by such energy balance interventions contributing to its anti-tumor effects [19]. Furthermore, modulation of the glucose metabolism also causes changes in signaling pathways such as phosphatidylinositol 3-kinase (PI3K)/Akt which is known to regulate numerous physiological processes in cancerous cells, contributing in tumorigenesis [20]. Also, the reduced insulin level by DER is known to decrease the hepatic synthesis of IGF-I, which regulates the progression of cell cycle from G1 to S phase through activation of PI3K/Akt signal transduction pathway [21, 22].

The glucose analogue 2-deoxy-D-glucose (2-DG) is a glycolytic inhibitor that is actively taken up by the cell via glucose transporters and phosphorylated (2-DG6-phosphate) by hexokinase, but not metabolized further by phospho hexose isomerase resulting in its trapping and accumulation inside the cell, causing the metabolic block [23]. In a glycolysis independent action, 2-DG disturbs N-linked glycosylation of proteins causing unfolded protein responses [24]. Collectively, it induces cytotoxicity as well as increase in radiation and chemotherapeutic drugs induced damage under *in vitro* and *in vivo* conditions, by inhibiting repair and recovery processes, besides augmenting cell death selectively in cancer cells [25, 26]. Interestingly, 2-DG spares or protects the normal cells and tissues from radiation and chemotherapeutic drugs induced damage by making it an ideal adjuvant in radio- and chemotherapy [27, 28]. Preferential cytotoxicity and enhancement in the treatment-induced death of cancer cells by 2-DG is contributed by a number of reasons like intracellular glucose deprivation, resulting in stress response such as metabolic oxidative stress [29] and inhibition of energy metabolism [26, 30]. More recent studies have shown that 2-DG in combination with focal irradiation of the tumor causes immune stimulation by way of activation of anti-tumor immunity in the peripheral blood, shift from Th2 and Th17 to Th1 linked to the depletion in T regulatory cells also contributes to the *in vivo* radio-sensitization [31]. Phase I-III clinical trials have shown that the orally administered 2-DG in combination with ionizing radiation is not only well tolerated, but provides survival advantage and better quality of life with negligible acute toxicity and protection to surrounding normal tissues [26, 32–34]. 2-DG has also been demonstrated to produce other beneficial effects as seen widely in DER models of several species at the systemic level [11, 35, 36].

Since metabolic reprogramming has been suggested to drive the process of carcinogenesis (and tumorigenesis), we have hypothesized that administration of 2-DG in an appropriate regimen may impair carcinogenesis (tumorigenesis) and therefore be a potential cancer preventive agent. Earlier studies have shown that dietary administration of 2-DG as a part of the feed inhibits methyl-1-nitrosourea (MNU) induced mammary carcinoma in rats [12]. Since 2-DG in the feed can result in hyperphagic response leading to higher food intake with further increase in 2-DG intake that may adversely affect the body growth, we considered feeding 2-DG in the drinking water. In the present studies, we have demonstrated that the chronic administration of 2-DG as a component of the diet can act as an ERMA that indeed significantly alters the process of tumorigenesis. Feeding of mice with 2-DG in drinking water for extended period of time significantly compromised the formation and growth of solid tumor from the implanted tumor cells in mice, while preserving the general physiological status of the mice to a very large extent as revealed by the body weight, blood profile (haematology), systemic glucose levels and immune status. Moreover, our observations also show that 2-DG mimics some of the effects of DER such as reduction in the core body temperature, serum insulin levels and tumor cell proliferation together with modulation seen in the host immune system and ECM. All of these together contributes to anti-tumor activity of 2-DG suggesting that factors contributing to the inhibition of tumorigenesis under conditions of classical DER can also be achieved by use of 2-DG as a potent ERMA.

## Materials & Methods

### Chemicals and cell line

The 2-deoxy-D-glucose (2-DG) was a gift from Dr. Reddy’s laboratory (Hyderabad, India). BrdU, Propidium iodide (PI), Ribonuclease-A (RNase-A) and 3, 5-Diaminobenzoic acid dihydrochloride (3, 5-DABA.2HCl) were obtained from Sigma Chemical Co., USA. Anti-BrdU primary antibody and FITC conjugated anti-mouse IgG_1_ secondary antibody were purchased from Becton Dickinson. Phosphate buffer saline (PBS), pepsin, saline (0.9% NaCl), HCl, BSA, Tween-20 and all other chemicals obtained were of analytical grade from BDH, Glaxo laboratories (Qualigens), SRL and E-Merck, India. Ehrlich’s ascites carcinoma (EAC) cells (strain F-3) obtained from Institute for Biophysics, University of Frankfurt, Germany were maintained by weekly intraperitoneal passaging in Swiss albino strain ‘A’ mice [25].

### Animals and dietary 2-DG administration

Female inbred Swiss albino strain ‘A’ mice (8–10 weeks old) weighing 22–28 g were obtained from the Institute’s central animal facility. Animal cages were maintained at 23–25°C with a 12-hour light/12-hour dark cycle and were fed standard rodent feed (from Golden Feeds, Delhi) and water *ad libitum*. Initially, a dose finding study was conducted to determine a maximum tolerable dietary dose of 2-DG that could be fed without appreciably affecting the normal physiology and animal growth but at the same time that could considerably inhibit the tumor growth. 50 female Swiss-Albino strain ‘A’ mice were randomized into ten groups fed with drinking water containing 0.0%, 0.02%, 0.04%, 0.06%, 0.08%, 0.1%, 0.2%, 0.4%, 0.6%, or 0.8% (w/v) 2-DG for 3 months. After 3 months of 2-DG treatment mice from all the groups were implanted with Ehrlich’s ascites carcinoma (EAC) cells on right flank and observed for tumor formation and growth. Optimally reduced body weights and significant tumor inhibition were the two dose determining end points. The dose optimization is well described in the results section and doses of 0.2% and 0.4% 2-DG were selected.

Thereafter, for further studies, mice were randomly assigned to one of the three groups: Control, 0.2% or 0.4% 2-DG (w/v). Mice in the 2-DG groups were fed with 2-DG containing daily drinking water with free access. However, 2-DG administration was continued beyond 3 months upon tumor implantation till the termination of the study as a more practical approach unlike in the dose finding study. Throughout the study, feed intake, water intake, body weight and temperature were measured.

### Ethics statement

Animal study was carried out in accordance with the recommendations in the Guide for the Care and Use of Laboratory Animals in cancer research of United Kingdom Coordinating Committee on Cancer Research (UKCCCR). The protocols used were approved by the Committee on the Ethics of Animal Experiments of the Institute of Nuclear Medicine and Allied Sciences (INMAS), Defence Research and Development Organization (DRDO) (Institutional Ethical committee number under which this study has been approved is INM/IAEC/2011/08/001). Mice were sacrificed by cervical dislocation with minimum suffering.

### Blood collection and biochemical analysis

Blood was directly withdrawn upon anaesthesia using ketamine/xylazine *(i*.*p)* from the retro-orbital plexus for peripheral blood mononuclear cells (PBMCs), serum and for haematology. Blood glucose was determined using ACCU-CHEK Active (Roche Diagnostics GmbH, Germany). Serum insulin levels were determined using the Ultrasensitive mouse insulin ELISA kit (Crystal Chem, USA). Haematology was performed using automatic haematology analyzer (MEK-6400) by Nihon Kohden (Japan) and data were generated using DMS-Lite software.

### Estimation of serum 2-DG levels

Under the above mentioned experimental conditions, serum levels of 2-DG were quantitated by fluorometric method which was a modification of the original procedure developed by Blecher [37]. This method is based on the acid-catalyzed condensation of 3, 5-Diaminobenzoic acid (DABA-2HCl) with 2-DG. Briefly, a working solution of 0.01 M DABA-2HCL was prepared in 5M Ortho-phosphoric acid. The standards (prepared in serum) and the samples were de-proteinized using 0.4 M Perchloric acid, incubated for 5 minutes at RT and centrifuged at 2000 RCF for 5 min at 25°C. 1 ml of supernatant was diluted with 2 ml of de-ionized water, mixed with 3 ml of 5 M DABA-2HCl, heated to 100°C in water bath for 15 min and then cooled at 15–17°C for 20 min in dark. Finally, 2 ml of 2.5M Ortho-phosphoric acid was then added to each test-tube and fluorescence was measured in the fluorescence microplate reader (Molecular Devices, USA) at an excitation wavelength of 420 nm and emission at 495 nm.

### Tumor Study

For tumor implantation, Ehrlich ascites carcinoma (EAC) cells (0.5 x 10^6^/100 μl PBS) were sub-cutaneously injected in the hind leg of mice (n = 14/group). Mice were observed for tumor latency, incidence, growth and survival. Six mice per group were sacrificed 28^th^ day post implantation (4 weeks) for further analysis and remaining 8 mice per group were observed for 60 days for tumor growth and survival. Briefly, the mice were regularly examined for palpable tumors and the tumor growth was measured every alternate day by electronic callipers. The mice were closely monitored for signs of tumor-related distress (lethargy, failure to thrive etc) once a tumor was detected. Mice were humanely sacrificed upon moribund condition, or after the tumors reached to a volume of 4500 mm^3^ as per the UKCCCR guidelines for the welfare of animals in experimental neoplasia [38]. Upon sacrifice the tumors were excised, weighed, and a part of it was kept immediately at -80°C for western blotting and zymography, and a part was fixed in 10% buffered formalin for histopathological evaluation. Single cell suspension by mechanical disruption was also prepared of a part of the tumor tissue for BrdU labeling for the proliferation assay. In addition, single cell suspension from spleens of these mice was prepared for immunostaining for further analysis.

### Tumor Imaging

To assess tumor burden non-invasively, the optical imaging was performed at day 21 post implantation. Near Infra Red (NIR) signals were recorded of dye that preferentially is taken up and accumulated by the tumor cells. Briefly, the mice were injected *i*.*p* with cyanine dye, IR-783 (Sigma) at a dose of 0.375 mg/kg body weight [39]. Whole body optical imaging was done 24 h later using In-Vivo F-PRO imaging station (Carestream Molecular Imaging, USA) station equipped with infrared fluorescent filter sets (excitation/emission-790/850 nm). Acquisition settings include 120 mm FOV, 2 × 2 binning, 1024 × 1024 pixel resolution, maximal gain and an exposure time of 10 seconds. Prior to imaging mice were anaesthetized with ketamine/xylazine. Data analysis was performed with Molecular Imaging Software 5.X (Carestream Molecular Imaging; USA).

### Measurement of tumor cell proliferation

To assess the effect of 2-DG on proliferation, the fraction of S phase cells, actively synthesizing DNA, were measured by flow cytometry using bromodeoxyuridine (BrdU) labeling assay. Briefly, single cell suspension was prepared from subcutaneous tumors (2 x 10^6^) and incubated with 10 μM BrdU in complete medium for 30 min, washed and fixed in 80% chilled ethanol and stored at 4°C. Immunostaining was performed as described earlier [40]. Flowcytometric analysis was carried out using LSR II flowcytometer (BD Biosciences, USA)

### Phenotyping of T cells

#### CD4, CD8 (splenocytes) subsets of T-cells

Single cell suspension of splenocytes was washed twice in PBS at 4°C, and 1 x 10^6^ cells were stained with antibodies (1:50, Cy5 conjugated-CD4 and PE conjugated-CD8 from eBioscience, CA) for 1 hr at 4°C. Following incubation, the cells were washed twice in PBS, acquired on LSR-II flow cytometer and analysed using FACS DIVA software (BD Bioscience, USA).

#### T-regulatory cells (peripheral blood)

PBMCs were isolated from whole blood after centrifugation over Ficoll–Histopaque (Sigma, St. Louis, USA) density gradient. For T-reg cells’ quantitation, the cells were incubated with Cy5 conjugated-CD4 and PE conjugated-CD25 antibodies (at 1:50 dilution; eBiosciences, CA) for 1 h at 4°C. Flowcytometric measurements and analysis was performed as described above.

### Western Blotting

Standard procedure was followed for western blotting as described previously [41] with some modifications. Briefly, tumor lysates were prepared by homogenization in lysis buffer [40 mM HEPES, 50 mM KCl, 1% Triton X-100, 1 mM Na_3_VO_4_, 50 mM NaF, 5 mM EDTA, 1 mM phenyl-methylsulfonyl fluoride, 1 mM benzamidine 1% Triton-X and complete protease inhibitor cocktail (Sigma)]. 40 μg of protein lysate per sample was loaded and resolved onto 10 or 12% SDS polyacrylamide gel and transferred to PVDF membrane. The levels of PI3K, Akt, phospho-Akt (Ser473), Glut-1, Hypoxia-inducible transcription factor-1α (HIF-1α) and β-actin were determined using specific primary antibodies (Santa Cruz Biotechnology, CA), followed by treatment with the appropriate peroxidase conjugated secondary antibodies. Immunoreactive bands were visualized using chemiluminescent reagent and captured using Microchemi (DNR Bioimaging Systems, Israel) gel documentation system.

### Gelatin zymography for MMP-9 activity

Gelatin zymography was used for analyzing the level of MMP-9 in the tumors. Briefly, the tumor tissues were homogenized in above mentioned lysis buffer without PMSF and protease inhibitor cocktail (PI). 50 μg of lysate sample was subjected to electrophoresis in 10% SDS-polyacrylamide gel containing 0.1% gelatin, under non-reducing condition. The gel was washed twice with washing buffer containing 50 mM Tris-HCl, pH 7.5, 2.5% Triton X-100 to remove SDS and then incubated overnight at 37°C in the reaction buffer (50 mM Tris-HCl, pH 7.5, 0.15 mM NaCl, 10 mM CaCl_2,_ 0.1% Triton X-100 and 0.02% NaN_3_). The gels were stained with 0.05% Coomassie blue-250 in solution (in methanol-glacial acetic acid-water; 5:2:5 v/v/v) and then destained with the same solution without the stain. The gelatinolytic activity was visualized as clear white zones against a blue background and photographed using MiniBIS Pro (DNR Bioimaging Systems, Israel). Densitometry was done with the DNR Gel Quant Express analysis software.

### Histology and immuno-histochemical (IHC) staining for PCNA

Tumor tissues were fixed in 10% neutral-buffered formalin (SRL) and embedded in paraffin. The tumor samples were sectioned at 5 μm thickness, stained with H & E (haematoxylin and eosin) at the Neurobehaviour laboratory (INMAS). For PCNA, sections were processed according to the manufacturer’s protocol (ImmunoCruz staining kit; Santacruz, CA). PCNA monoclonal primary antibody (Santa Cruz Biotechnology, CA) was used at 1:100 dilution; overnight at 4°C. Corresponding tissue sections without primary antibody served as negative controls. The sections were examined by light microscopy to capture bright-field images using Olympus (IX51) microscope.

### Statistical Analysis

All the data were analyzed using GraphPad Prism (version 5.01) and are presented as mean ± standard error of the mean (SEM). For statistical significance One-way or two-way ANOVA was used with Tukey’s or Dunnett’s or Bonferroni’s multiple comparison post test. Latency and survival of tumor bearing mice were analyzed using the Kaplan-Meier method with log rank test for significant differences.

## Results

### 2-DG dose optimization in Swiss albino strain ‘A’ mice

**Fig 1:**
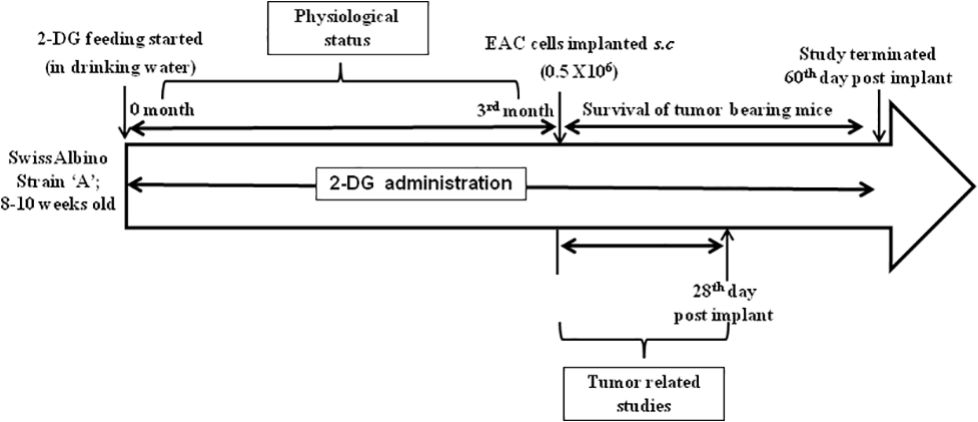
Experimental design to evaluate the tumor inhibitory effect of dietary 2-DG. Mice were administered with 2-DG in the daily drinking water for 3 months prior to tumor implantation and continued till the termination of the study.

In order to assess the effect of the glycolytic inhibitor 2-DG in mimicking DER and inhibiting tumorigenesis, adult Swiss albino strain ‘A’ mice were administered with 2-DG in their daily drinking water. For tumor implantation, we employed the EAC tumor model as it mimics the clinical presentation (undifferentiated, rapidly growing, and highly malignant with no tumor specific transplantation antigen) [42]. Optimization of the dose was carried out by investigating both body weight changes and tumor formation with a wide range of 2-DG (0 to 0.8% w/v) fed continuously for three months and discontinued before implantation of tumor cells. The total body weight gain at the end of the study (3months of 2-DG feeding) was either relatively higher at lower doses of 0.02%, 0.04% and 0.06% w/v 2-DG or similar at 0.08% w/v 2-DG to the control (0.0%) mice, with tumor formation essentially similar (100%) to unfed mice (Table 1). Therefore, these doses were not selected since the objective was to have slight reduction in body weight along with significant tumor inhibition. At doses of 0.1, 0.2 and 0.4% w/v 2-DG, the body weight gain was slightly decreased (< 20%) in comparison to the control mice with a tumor incidence of 80%, 60% and 60% respectively. On the other hand, at higher doses of 0.6% and 0.8% w/v 2-DG, 20% and 40% mortality respectively with significant reduction in body weight were noted during the 3 months period of 2-DG and therefore discontinued before tumor implantation. Based on these observations, we selected doses of 2-DG (0.2% and 0.4% w/v) that resulted in mild decrease in body weight and reduced tumor formation. For further studies, 2-DG was given for 3 months prior to tumor implantation and thereafter continued till the termination of the study according to the experimental protocol summarized in Fig 1A.

**Table 1:** 2-DG dose optimization in Swiss albino strain ‘A’ mice. Total body weight gain (final; day 90 –initial weight; day 0) and EAC tumor formation (n = 5/group).

Groups	Total weight gain (g)	Tumor incidence
Control	3.0 ± 0.27	5/5 (100%)
0.02% 2-DG	5.8 ± 0.4	5/5 (100%)
0.04% 2-DG	3.8 ±0.51	5/5 (100%)
0.06% 2-DG	3.5 ± 0.31	5/5 (100%)
0.08% 2-DG	3.0 ± 0.27	5/5 (100%)
0.1% 2-DG	2.7 ± 0.4	4/5 (80%)
0.2% 2-DG	2.6 ± 0.1	3/5 (60%)
0.4% 2-DG	2.5 ± 0.55	3/5 (60%)
0.6% 2-DG	2.25 ± 0.52	-
0.8% 2-DG	2.33 ± 0.16	-

### Dietary 2-DG does not adversely affect the physiology of the mice

**Fig 2:**
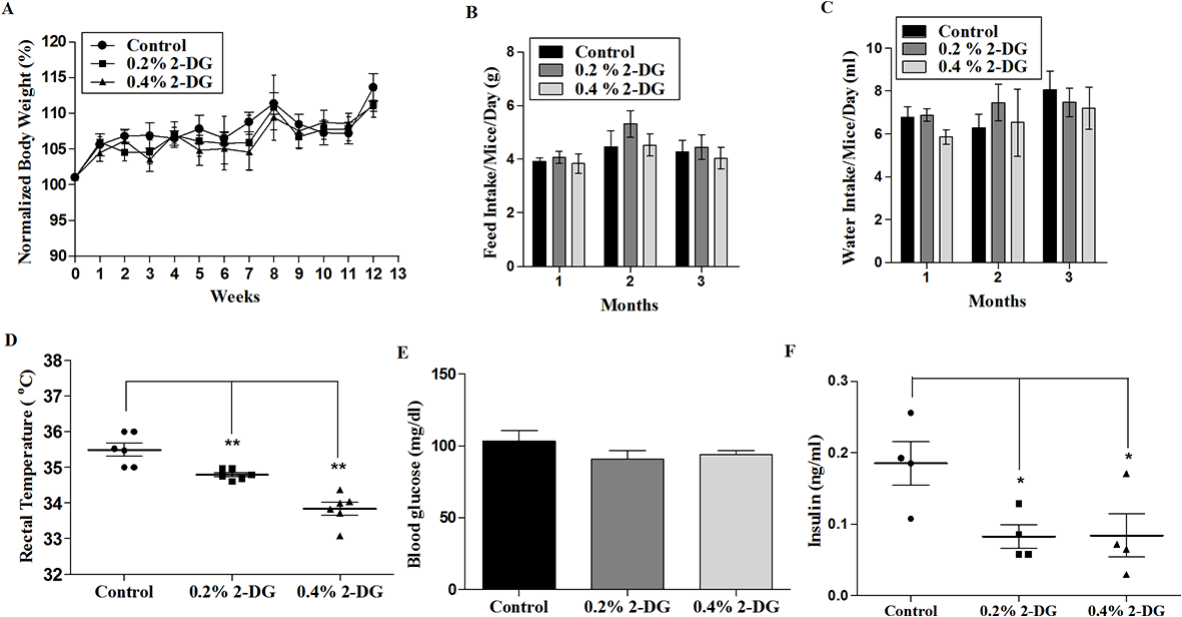
Effect of dietary fed 2-DG on general physiological parameters in mice. (A) Body weight (normalized), (B) Feed intake, (C) Water intake, (D) Body temperature, (E) Blood glucose and (F) Serum insulin levels. Body weight, feed intake, water intake were measured weekly for 3 months of 2-DG feeding, whereas body temperature (anal) was measured at the end of 3 months. Blood glucose and serum insulin levels were analysed at day 60 of the 3 months period. Values presented are means ± SE. *, p < 0.05; **, p < 0.01; ***, p < 0.001

To determine the effect of dietary 2-DG administration (0.2% and 0.4%) on the growth of mice, body weight of the mice was measured regularly throughout the study. Mice fed with 2-DG had marginally reduced body weights at both doses compared to the control group (Fig 2A). The average body weight gain after 3 months were 2.75 ± 0.42 and 2.66 ± 0.21 grams in the 0.2% and 0.4% 2-DG groups respectively, compared to 3.25 ± 0.44 grams in control (Table 2), which is 15% (p = 0.51) and 18% (p = 0.45) lower respectively, than the control. The average feed and water intake was also not significantly changed by 2-DG under these conditions (Fig 2B and 2C). It is important to note that the feeding of 2-DG resulted in marginal decrease in body weight (non significant), although the feed intake was similar to that of untreated animals. A dose dependent decrease in the rectal temperature was also observed in 2-DG fed mice (Fig 2D); the mean rectal temperature at 3 months of 2-DG administration was 34.79°C (p < 0.05) and 33.84°C (p < 0.001) in the 0.2% and 0.4% 2-DG groups respectively compared to 35.47°C of the control group.

**Table 2:** Body weight changes of mice after 3 months of 2-DG administration (0.2% and 0.4% w/v in daily drinking water).

	Control	0.2% 2-DG	0.4% 2-DG
Initial weight (g)	26.0±0.46	27.41±0.99	26.58±0.78
Final weight (g)	29.25±0.35	30.16±0.8	29.25±0.85
Total weight gain (g)	3.25±0.44	2.75±0.42	2.66±0.21
Wt. gain/day/mice (g)	0.036±0.005	0.031±0.005	0.030±0.002

Note: Initial and final weights are mean body weights at day 0 and day 90 respectively. Data are expressed as mean ± SE. Statistical analysis (One-Way ANOVA) indicated a non-significant reduction in the body weights of 2-DG administered mice at both the doses.

The levels of blood glucose and serum insulin were assessed during the course of the study. A marginal reduction in the blood glucose levels was observed (shown at day 60) during the 3 months period of 2-DG administration (Fig 2E). Interestingly, the insulin concentration in the serum was significantly reduced in 2-DG fed mice (Fig 2F), measured at day 60 (0.082 ±0.016 and 0.084 ± 0.03 in 0.2% and 0.4% 2-DG respectively versus 0.18 ± 0.30 ng/ml in control; P < 0.05 for both the doses).

The haematological parameters suggest that 2-DG does not alter erythrocyte number, haemoglobin content and platelets number, whereas a slight increase was observed in total leukocyte, lymphocytes and granulocytes numbers (S1 Table). Most importantly, all values of the haematological parameters were within the physiological range.

### Levels of circulating 2-DG

**Fig 3:**
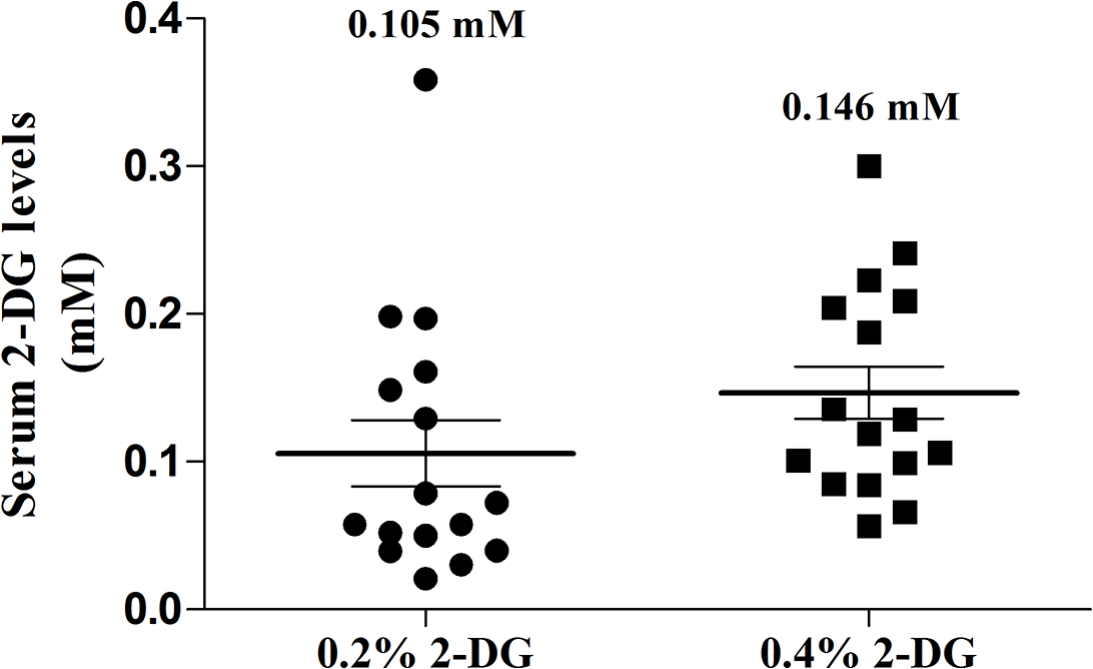
Circulating levels of 2-DG. The 2-DG concentration in the serum of mice administered with drinking water containing 0.2% and 0.4% 2-DG (w/v). No traces of 2-DG were found in the control animals as expected. 2-DG levels were estimated fluorimetrically based on the acid catalyzed condensation of DABA with 2-DG, with some modifications in the method described by Blecher (44). For detailed protocol, see Material and Methods.

The effective serum levels of 2-DG were estimated in mice fed with 2-DG from blood collected on random days at 11:00 am or 3:00 pm considering the variation of the feeding and water intake habits. The mean 2-DG concentration was found to be 0.105 ± 0.022 and 0.146 ± 0.017 mM in serum of 0.2% and 0.4% 2-DG (w/v) fed mice respectively (Fig 3). These are equivalent to 1.722 mg/dl in 0.2% and 2.39 mg/dl in 0.4% 2-DG group resulting in 2-DG to glucose ratio of 0.018 and 0.025 respectively. As such no differences were found in 2-DG concentration in serum collected at 11 am or 3 pm.

### Dietary 2-DG lowers the incidence and reduces the growth of implanted EAC tumors

**Fig 4:**
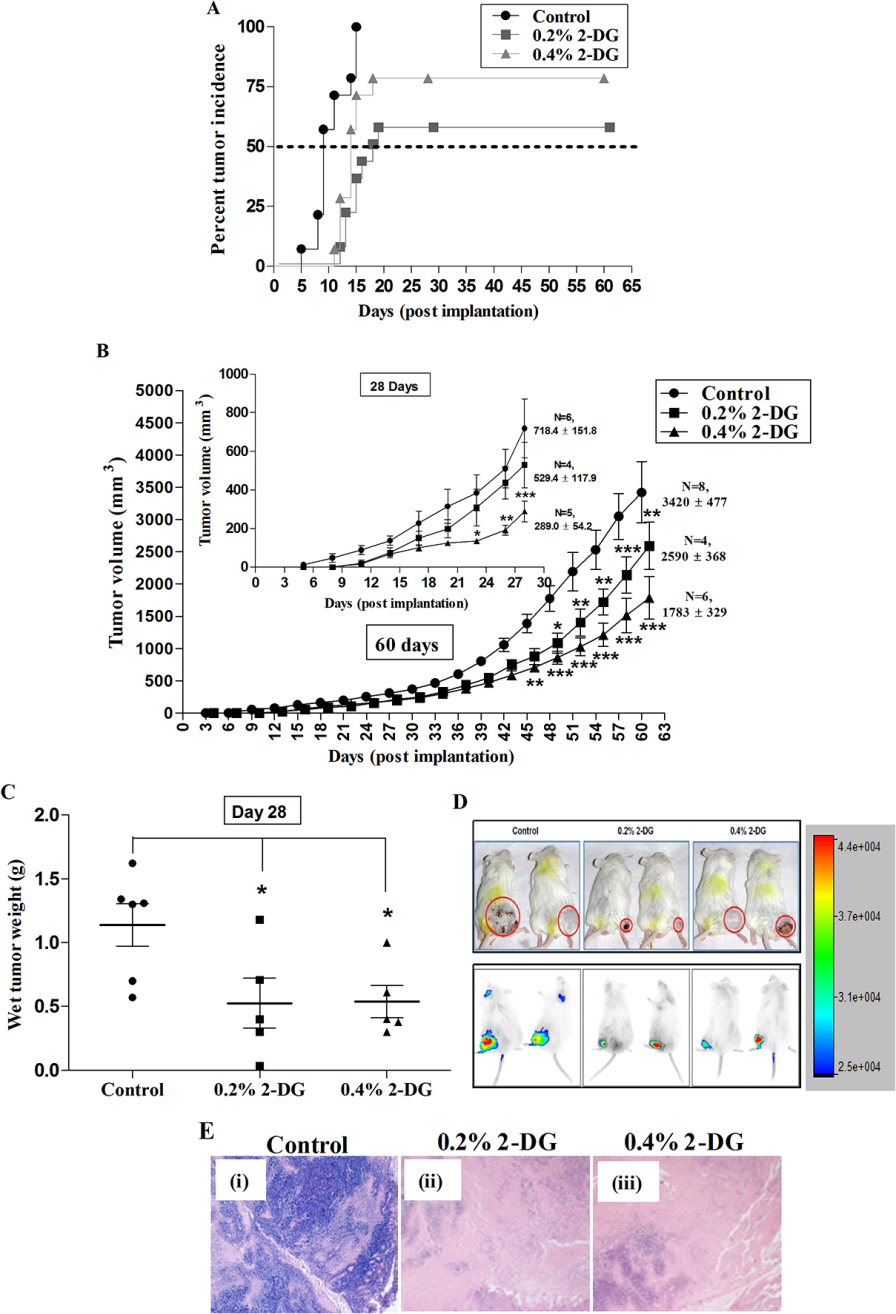
Dietary 2-DG significantly inhibited the process of tumorigenesis in transplanted Erhlich ascites carcinoma (EAC) tumor model. (A) Tumor latency (palpable) and incidence, (B) Tumor volume, (C) Tumor weight (day 28), (D) Representative photographs of animals showing differences in the tumor size (upper panel) and in-vivo optical images (lower panel). Intensity of the signals showing the NIR-dye tumor specific uptake in mice from each treatment group demonstrating tumor burden (day 28), (E) Representative photomicrographs of histological analysis (H & E staining) showing intra-tumoral morphology of EAC tumors from (i) Control, (ii) 0.2% 2-DG and (iii) 0.4% 2-DG mice. *, p < 0.05; **, p < 0.01; ***, p < 0.001

The tumor implantation was carried out to investigate the effects of 2-DG on the process of tumorigenesis. Dietary 2-DG lowered the overall incidence of EAC tumors by nearly 40% and 25% at doses 0.2% and 0.4% respectively (Fig 4A). In addition, 2-DG significantly delayed the onset (median days) of palpable tumors by 9 days (9 vs 18 days) and 5 days (9 vs 14 days) in 0.2% and 0.4% groups respectively (Fig 4A; Log-rank test: p < 0.05). Tumor development was assessed at 2 post-implantation time points (Fig 4B); one at 28^th^ day post implant (inset), where a reduction in the average tumor volume of 26% (p > 0.05; non-significant) in 0.2% 2-DG group and 60% (p < 0.001) in 0.4% 2-DG group was observed. The wet weight of tumors in 2-DG fed mice was also significantly lower at both doses, with the mean tumor weight of 0.52 ± 0.19 g (p < 0.05) and 0.54 ± 0.12 g (p < 0.05) in 0.2% and 0.4% 2-DG respectively versus 1.14 ± 0.16 g in control (day 28, Fig 4C). In the second one at 60 days post-implant, there was a 24.2% (P < 0.01) and 47.8% (P < 0.001) reduction in the tumor volume in 0.2% and 0.4% 2-DG groups respectively. Representative photographs depicting the gross tumor size differences observable between the 2-DG fed and the control mice are shown in Fig 4D (upper panel). Also, the tumors from 2-DG fed mice exhibited lower tumor neovascularisation (vascular density) than the tumors of the control mice (S1 Fig).

Tumor burden was also assessed by non-invasive near-infrared (NIR) fluorescence in-vivo imaging at day 21 using heptamethine cyanine dye, IR-783, with preferential uptake and retention in tumor cells. Images revealed relatively lower signals from the tumors of the 2-DG fed groups compared to higher signals from the tumors of the control group (Fig 4D; lower panel), which correlated well with the tumor volume measured using caliper. The morphological examination showed sparse distribution of tumor cells in 2-DG fed mice, as compared to diffused and ill-defined spread of cells seen in untreated animals (Fig 4E).

### Dietary 2-DG reduces tumor cell proliferation

**Fig 5:**
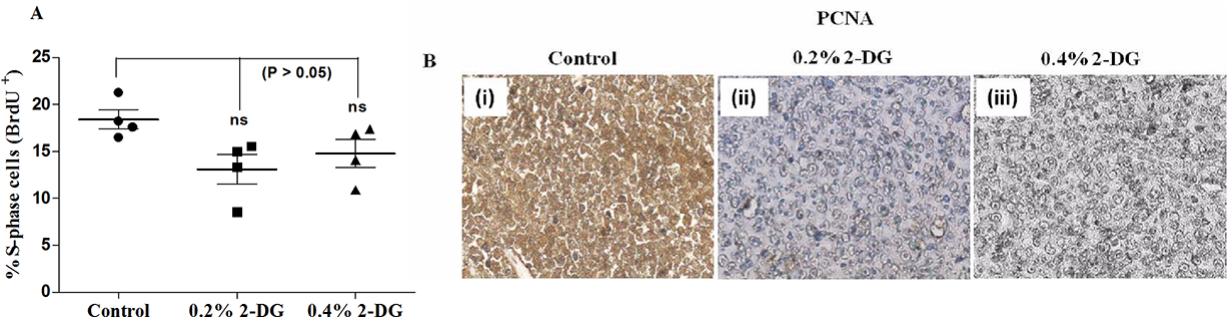
Effect of dietary 2-DG on tumor cell proliferation in EAC tumor model. (A) BrdU positive (proliferating) cells analyzed by flow cytometry. S-phase cells in tumors of 2-DG fed mice were numerically reduced, although the differences were not statistically significant (One-way ANOVA), (B) Immuno-histochemical staining of PCNA in tumors; (i) Control, (ii) 0.2% 2-DG and (iii) 0.4% 2-DG. Tumors from 2-DG treated animals exhibited reduced PCNA staining in comparison to control group animals.

To investigate if the reduction in tumor growth induced by 2-DG administration is due to inhibition of tumor cell proliferation, we analysed the presence of S-phase cells using BrdU pulse labelling. The percentage of cells staining positive for BrdU was numerically decreased (statistically non-significant; p = 0.06) from approximately 18% ± 1.0% in control mice to 13% ± 1.5% and 14% ± 1.4% in 0.2% and 0.4% 2-DG fed mice respectively (Fig 5A). The 2-DG group tumors, relative to control tumors, had significantly reduced cellular proliferation (PCNA staining; Fig 5B), which corresponds to the BrdU assay results suggesting a decrease in the proliferation of tumor cells by 2-DG.

### Dietary 2-DG enhances survival of tumour bearing mice

**Fig 6:**
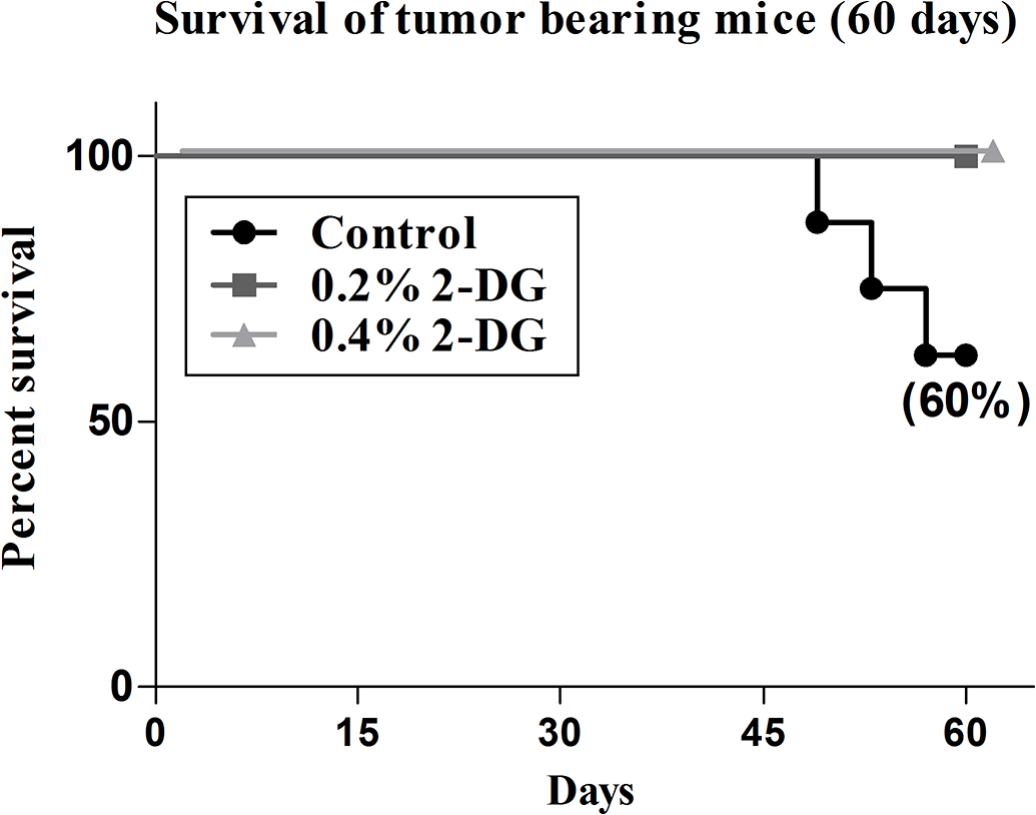
2-DG administration significantly enhanced the survival of EAC tumor bearing mice. Kaplan-Meier plot showing differences in the tumor burden related death observed till day 60 post implant. The Log rank analysis of the survival plot showed a significant increase in the lifespan of tumor bearing mice fed with 2-DG (p < 0.05) at both the doses.

Since many of the beneficial effects of DER were observed in 2-DG fed mice, we also evaluated whether long-term 2-DG administration could extend lifespan of the EAC tumor bearing mice. This survival study lasted only for 60 days and mice were sacrificed thereafter because of the tumor related discomfort to the mice especially in the control group, as per the UKCCCR guidelines [38]. The Log rank analysis of the Kaplan-Meier survival plot (Fig 6) showed significant increase in the lifespan of tumor bearing mice with 100% survival of the 2-DG fed mice (at both the doses) compared to 62.5% in control group (60 days; p < 0.05). Collectively, these results show that 2-DG significantly reduced the tumor incidence, tumor growth, delayed the onset and improved the survival of mice bearing the EAC tumor.

### Effect of dietary 2-DG on CD4^+^, CD8^+^ T-cells and CD4^+^CD25^+^ T-reg cell populations

**Fig 7:**
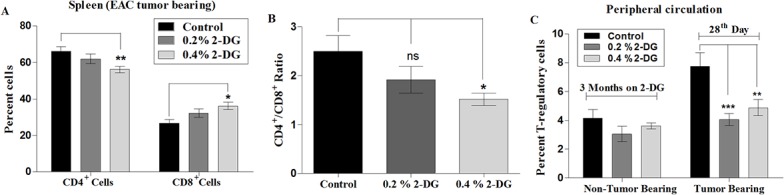
Effect of 2-DG on T-cell populations. (A) 2-DG had a differential and dose dependent effect on CD4^+^ (decrease) and CD8^+^ (increase) T-cell subpopulations reflected in reduced (B) CD4^+^/CD8^+^ ratios. Lymphocytes from the spleen of control and 2-DG fed EAC tumor bearing mice were analyzed by flowcytometry to quantitate percentages of CD4^+^ and CD8^+^ populations. Also the effect of 2-DG administration on the (C) CD4^+^CD25^+^ Treg cell population was determined in the peripheral circulation of non tumor bearing and EAC tumor bearing host by flowcytometry. 2-DG prevents tumor-induced augmentation of CD4^+^CD25^+^ Treg cells in tumor-bearing mice compared to no alteration in the non tumor bearing mice. *, p < 0.05; **, p < 0.01; ***, p < 0.001

Since immune status plays an important role in modulating the growth of tumors, we investigated the effects of 2-DG on the splenic population of CD4, CD8 and circulatory T-regulatory cells (PBMCs) of the mice. The percentage of splenic helper T lymphocytes (CD4^+^) decreased in a dose dependent manner (61.9% ± 2.5%; p > 0.05 and 56% ± 1.7%; p < 0.01 in 0.2% and 0.4% 2-DG respectively compared to 66% ± 2.7% in control mice), whereas the cytotoxic T lymphocytes (CD8^+^) showed a dose dependent increase (32% ± 2.3%; p > 0.05 and 36% ± 2.0%; p < 0.05 in 0.2% and 0.4% 2-DG respectively versus 26% ± 1.9% in control; Fig 7A). These changes were clearly reflected in the altered CD4^+^/CD8^+^ ratio (Fig 7B). The fraction of T-regulatory (T-reg) cell population was significantly elevated in the peripheral circulation of tumor-bearing mice when compared to that of normal mice (Fig 7C). Interestingly, 2-DG prevented tumor-induced augmentation of CD4^+^CD25^+^ T-reg cells in tumor bearing mice (Fig 7C; 4.07% ± 0.41%; p < 0.001 and 4.89% ± 0.55%; p < 0.01 in 0.2% and 0.4% 2-DG group respectively compared to 7.74 ± 0.95 in control). In the non-tumor bearing animals, 2-DG did not alter the T-reg population (Fig 7C).

### Dietary 2-DG improves glucoregulation and reduces PI3K/Akt signalling

**Fig 8:**
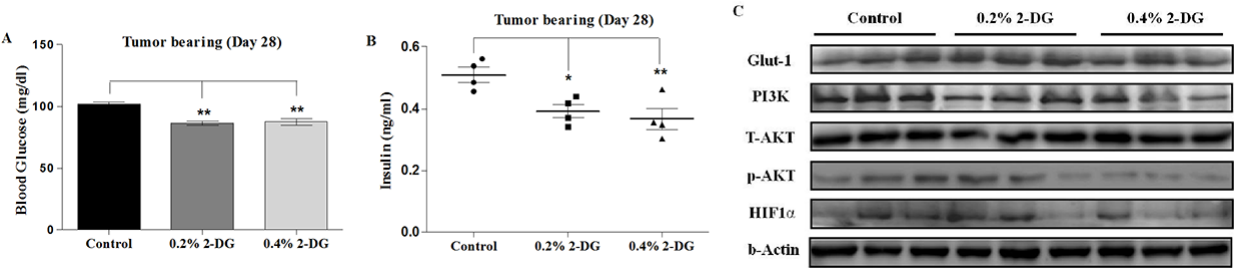
Dietary 2-DG reduces blood glucose, serum insulin and PI3K/AKT signalling. 2-DG administration reduces (A) blood glucose, (B) serum insulin in EAC tumor bearing mice (day 28) as observed in the non-tumor bearing mice shown in Fig 2E and 2F), and down-regulated (C) PI3K/Akt signalling in tumors. Western blot results revealed reduced expression of PI3K, phosphorylated-Akt and HIF-1α. No changes were observed in the expression levels of Glut-1 and T-Akt. β-Actin was used as loading control.

We further examined the effects of 2-DG on the levels of certain metabolism related molecules which play important roles in tumor metabolism and growth. A significant reduction in blood glucose and serum insulin levels was observed in the tumor bearing mice, fed with 2-DG compared to the control mice. The mean blood glucose levels measured at day 28 post tumor implantation were 86.5 ± 1.7 and 87.5 ± 2.7 mg/dL at 0.2% and 0.4% doses of 2-DG (w/v) compared to 101.7 ± 1.6 mg/dL (p < 0.05) in control mice (Fig 8A). Similarly, the serum insulin levels were also considerably lower in the 2-DG fed mice in comparison to the control in tumor bearing condition (0.39 ± 0.02 and 0.36 ± 0.03 ng/ml for 0.2% (p < 0.05) and 0.4% (p < 0.01) 2-DG groups respectively versus 0.5 ± 0.02 ng/ml for the control group; Fig 8B). Consistently, 2-DG, in non-tumor as well as tumor bearing mice, reduced the blood glucose and insulin levels suggesting an improved glucoregulation or better glucose homeostasis.

Increased expression of insulin and IGF-1 receptors in the pre-neoplastic and neoplastic cells leading to up-regulation of insulin signaling is known to be involved in the progression of tumorigenesis through activation of PI3K/Akt cascade. Western blot analysis (Fig 8C) revealed that the expression of PI3K, phosphorylated Akt (S-473) and HIF-1α was significantly lower in the EAC tumor tissue of the 2-DG fed mice than in the tumors of control mice. 2-DG had no significant effect on expression of Glut-1 and T-Akt. Taken together, these results indicate that the 2-DG induced reduction of Akt (S-473) phosphorylation in EAC tumors could be associated with the decrease in the insulin levels.

### Dietary 2-DG reduces tumor invasiveness

**Fig 9:**
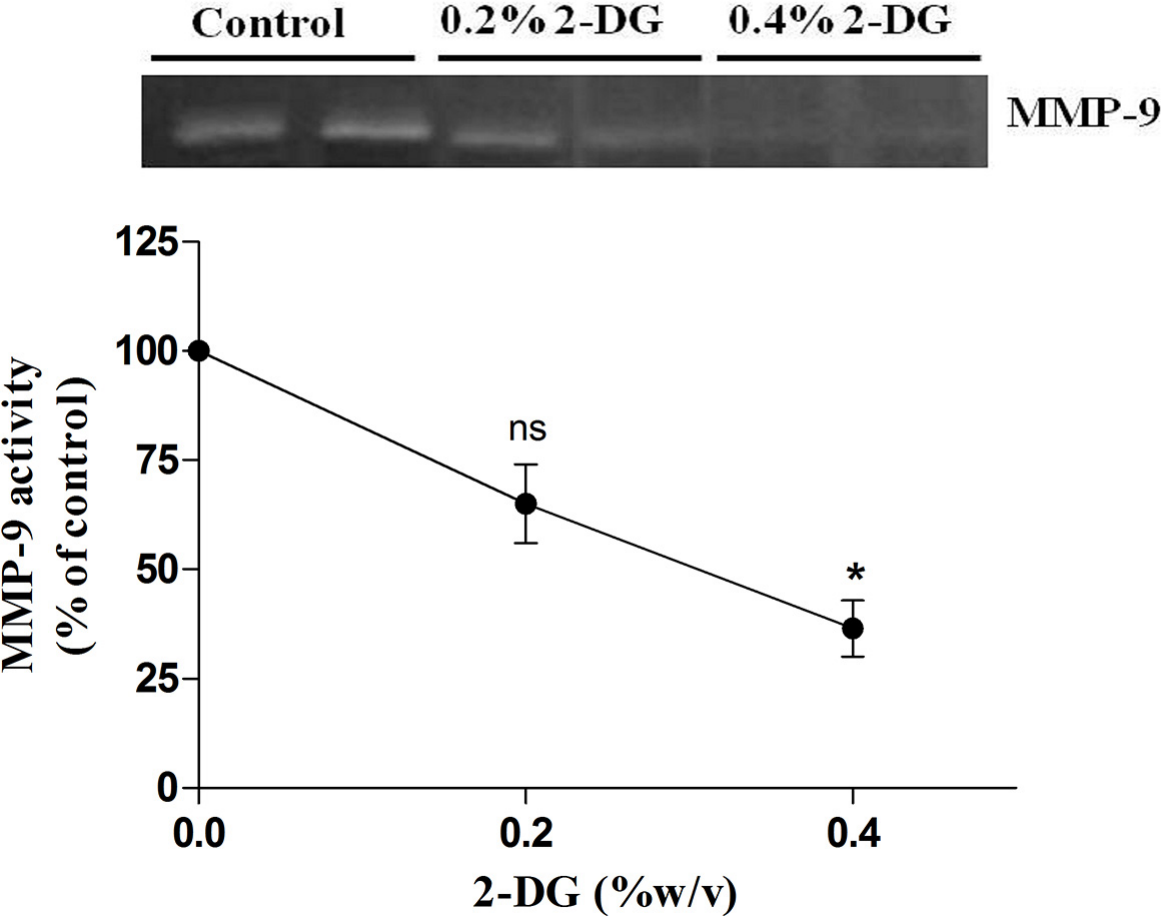
2-DG administration reduces MMP-9 activity. A dose dependent decrease was observed in the gelatinolytic activity of the EAC tumor tissue lysate from 2-DG fed mice in comparison to control mice. Scanning densitometry was used to quantify the MMP-9 gelatin hydrolysis (zymography) as shown in the graph. Data shown are representative of two independent experiments.

Matrix metalloproteinases (MMPs) are known to be involved in the degradation of the extracellular matrix facilitating tumor cell migration and local invasion along with activation of vascularisation in the tumor stroma [43]. Therefore, we evaluated the expression of MMP-9 specifically, by gelatin zymography, in the tumor tissue lysate based on its enzymatic activity. Quantification of the intensity of the MMP-9 specific bands (zone of clearance) showed a significant reduction of 35% and 63% (in 0.2% and 0.4% 2-DG groups respectively) in the gelatinolytic activity of MMP-9 in comparison to the control group (Fig 9). These results suggest that dietary administration of 2-DG does alter tumor associated signal transduction events such as ECM modulation controlling MMP-9 secretion and therefore tumor growth and invasion.

## Discussion

DER is a very potent and broad acting strategy against a number of diseases including cancer and experimental evidences suggest that the anticancer effect of DER is mainly because it targets up-regulated glycolysis (Warburg’s effect) of the tumors [4, 5, 12, 19]. In humans, DER is practically difficult to follow as it involves a reduction in the actual dietary or calorie intake and therefore, other strategies are being explored such as the use of ERMAs [8–12, 35, 44]. Here we show that dietary supplementation of the glycolytic inhibitor; 2-DG in mice has effects that in many aspects mimic the DER effects thereby effectively inhibiting the process of tumorigenesis.

Continuous exposure to 2-DG at higher doses leading to prolonged accumulation, is known to cause glucopenia and disruption of thiol metabolism resulting in cytotoxicity [45, 46]. However, the doses used in the current studies were much lower than the therapeutic doses shown to be effective as an adjuvant in the radio-sensitization of tumors [33], yet significantly inhibited the process of tumorigenesis. It is pertinent to mention that in the earlier studies designed with 2-DG either as a therapeutic or adjuvant it was administered either as a single dose or multiple doses. In the present studies, we could achieve a sustained serum 2-DG concentration of 0.1 to 0.15 mM (Fig 3) when given in daily drinking water. These concentrations are considerably lower than the levels achieved in plasma when 2-DG is administered at therapeutic doses as well as concentrations found to be effectively cytotoxic under *in-vitro* conditions. This indicates that sustained low levels of 2-DG for chronic period may cause modulation of certain host factors. Comparable 2-DG blood levels were observed (0.2 mM) in a recent clinical trial for the treatment of prostate cancer where the 2-DG dose administered was 30 mg/kg body weight [47] suggesting that it is easily achievable in humans. Importantly, we did not observe any overt pathology in 2-DG fed mice suggesting safety and feasibility of such metabolic intervention. Indeed, our study revealed that 2-DG supplementation mimics some effects similar to DER as discussed below suggesting use of dietary interventional agents such as 2-DG as a suitable alternative to DER. We observed a 15–18% decreased body weight in 2-DG fed mice compared to the control (Table 2 and Fig 2A), which has been described as a moderate caloric restriction in rodents [36]. In addition, the mice maintained on 2-DG did not exhibit any hyperphagic response as suggested by lack of increase in their food intake (Fig 2B), although hyperphagia was known to occur as a compensatory mechanism with DER [48]. Also the core body temperature was found to be reduced in 2-DG fed mice (Fig 2D), similar to what is observed in organisms under conditions of such metabolic stress [36]. Since 2-DG is a known metabolic stressor, this fall in body temperature can be described as an adaptive response in order to decrease the overall metabolic rate and to conserve energy by allowing less loss of energy in the form of heat [36, 49]. Optimal decreases in body weight and temperature as observed in our study are important suggested markers of energy restriction-like condition.

In the tumor implant model system (Ehrlich’s ascites carcinoma) used here, we employed minimum number of cells (0.5 X 10^6^, tumor take number) which could mimic initiation stage of tumorigenesis. Dietary 2-DG appreciably decreased the tumor formation (incidence) in the first place along with significant delay in the onset (palpable tumors; Fig 4A) with a slower tumor growth rate (Fig 4B). These findings suggest that dietary 2-DG impairs the process of tumor formation rather than compromising only the tumor growth. As expected, this resulted in prolonged survival of tumor bearing mice suggestive of reduction in the tumor related death due to reduced tumor burden (Fig 6).

During the three months period of 2-DG administration prior to tumor implantation, we observed a marginal decrease in the blood glucose level with a significant reduction in serum insulin concentration indicating increased insulin sensitivity (Fig 2E and 2F). This suggests improved glucoregulation achieved by dietary 2-DG without reducing the dietary intake similar to what is reported in dietary restriction condition [50]. Chronic hyperinsulinemia is known to increase the risk of cancer predisposition especially in metabolic syndromes [51]. Enhanced insulin level upregulate the synthesis of IGF-1 in the liver known to promote proliferation by facilitating the progression of cells from G1 to S-phase with reduced apoptosis [13, 52]. Dietary 2-DG could also reduce the insulin levels in the tumor bearing mice (Fig 8B), where the insulin levels in the tumor bearing control mice increased > 2 folds compared to the non tumor bearing control mice. Decrease in insulin levels, may be partly responsible for the anti-tumor effect, thereby reducing the rate of tumor growth as observed from the reduced BrdU and PCNA staining in the tumors of 2-DG fed mice (Fig 5A and 5B), indeed supporting this proposition.

PI3K/Akt pathway is one of the key metabolism linked mediators which provides tumor cells with proliferative and antiapoptotic advantage and DER has been shown to perturb this pathway [20]. The PI3K/Akt signal transduction pathway gets activated upon activation of receptor tyrosine kinases (RTK) which are known to get activated by receptor binding of insulin and/or IGF-1 [53]. Also, the HIF-1α is a major downstream effector of Akt that regulates glucose flux and metabolism as shown in various tumors exhibiting Warburg effect [54]. In the present study, we found that tumors of 2-DG fed mice had decreased levels of PI3K, phosphorylated Akt, and HIF-1α (Fig 8C), which was in line with other studies showing down-regulation of PI3K/Akt signalling contributing to the anti-tumor effect of DER in murine tumor models. This reduction in the tumor growth by 2-DG could be, in part, due to reduced insulin levels leading to attenuation of the insulin/IGF-1/PI3K/Akt/HIF-1α signaling pathway thereby possibly modulating the various cell cycle regulatory proteins (cyclin D, A, E) involved in the proliferation [55, 56].

Notably, the tumors from 2-DG fed mice showed marked reduction in their vascular networks (S1 Fig) that could be partly attributed to reduced MMP-9 activity (Fig 9). Tumor-secreted MMP-9 activity is known to activate the proangiogenic factor, vascular endothelial growth factor (VEGF) and favours tumor invasion as well as tumor associated neovascularization [57, 2]. The exact mechanism by which 2-DG reduces MMP-9 activity is not clear, but it may be responsible for the reduction in tumor invasiveness and associated angiogenesis.

Emerging evidences suggest that the immune system is also an important factor contributing to the protective features of DER and fasting [58, 59]. It is very well established that the tumors try to escape the host antitumor immune response; one such mechanism is by suppression of CD8^+^ effector T-cells and enhancing Tregs suppression activity [60, 61]. While CD8^+^ (CTLs) cells are known to play a role in antigen-specific tumor killing, T-regs regulate the antitumor immune function by suppression of effector CD8^+^ (CTLs) cells [62]. In the present study we found a dose dependent decrease in CD4^+^ to CD8^+^ T-cell ratio (Fig 7A and 7B) in 2-DG fed mice suggesting an enhanced antitumor immunity. Also, 2-DG significantly prevented the tumor induced augmentation of T-regulatory cells (CD4^+^CD25^+^; Fig 7C) which corresponds to increase in CD8^+^ (CTLs) cells. Lack of any effect on the levels of CD4^+^ and CD8^+^ as well as the T-reg population in the non-tumor bearing mice suggest that dietary 2-DG modulates the immune alterations brought about by growing tumors.

Shift in the glucose metabolism in majority of the cancer cells from oxidative phosphorylation to aerobic glycolysis (Warburg effect) provides us with the opportunity to target this metabolic reprogramming for controlling tumor growth. There are various approaches for carrying out energy restriction such as dietary restriction (reduced dietary intake), caloric restriction (like carbohydrate and/or fat content alterations) or alternate day fasting (ADF). However, use of ERMAs provides with much better approach to target tumor without actually cutting down the dietary intake. The exact mechanisms by which DER reduces tumour growth are not yet clear, but it is likely that they involve multiple factors in tumour cells as well as host cells. Since majority of tumors depend on glucose as their main energy source, such tumours should be responsive to nutrition interventional strategies.

In conclusion, these effects of dietary 2-DG on blood glucose, insulin, PI3K/Akt signaling, proliferation, immune status and MMP-9 activity provide a plausible mechanism for the tumor inhibitory effects of such metabolic intervention. This study provides information about the likely involvement of multiple factors and potential molecular targets in the inhibition of the process of tumorigenesis by use of such nutritional interventional agents as a practical suitable alternative to DER.

## Supporting Information

S1 Fig 2-DG fed mice showed reduced tumor associated angiogenesis. Representative photographs showing reduced microvasculature (vascular density) in EAC tumors of 2-DG fed mice in comparison to tumors from control group of mice. Red arrows indicate the extent of vascularity.
(TIF)

S1 Table Effect of dietary 2-DG on the blood indices of mice. Blood hematology was carried out after 3 months of 2-DG feeding before tumor implantation.
(DOC)
