# Supplementary figures and images for: Chronic Dietary Administration of the Glycolytic Inhibitor 2-Deoxy-D-Glucose (2-DG) Inhibits the Growth of Implanted Ehrlich’s Ascites Tumor in Mice
Source: PLoS One. 2015 Jul 2;10(7):e0132089. doi: 10.1371/journal.pone.0132089 (PMC4489743; doi:10.1371/journal.pone.0132089)

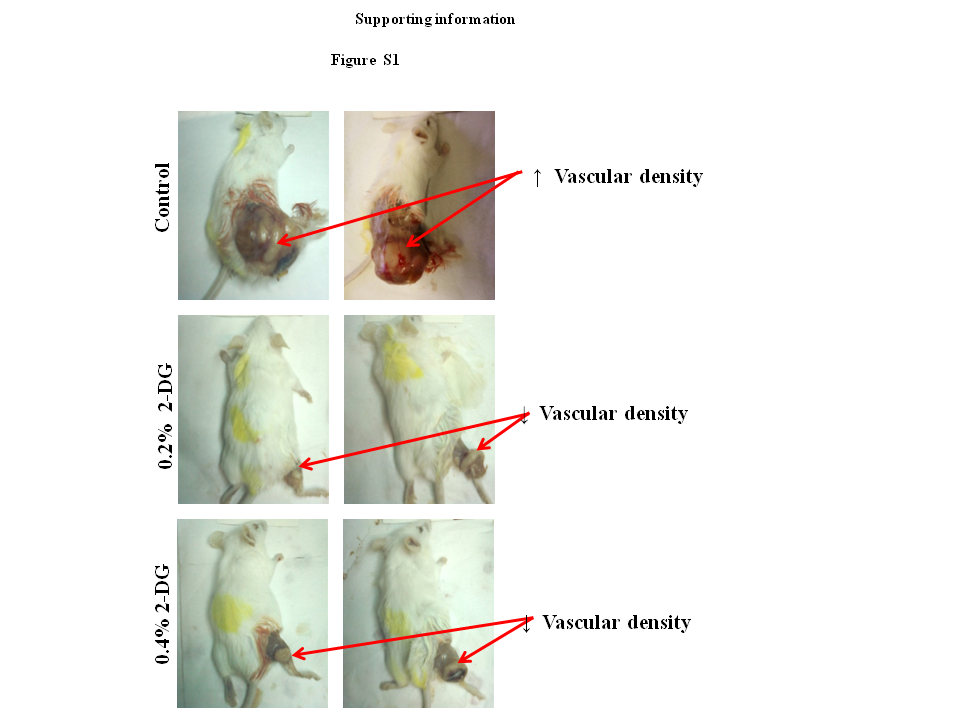

Supplement: S1 Fig — Representative photographs showing reduced microvasculature (vascular density) in EAC tumors of 2-DG fed mice in comparison to tumors from control group of mice. Red arrows indicate the extent of vascularity. (TIF) [file pone.0132089.s001.tif]
