# Supplementary material for: Chronic Dietary Administration of the Glycolytic Inhibitor 2-Deoxy-D-Glucose (2-DG) Inhibits the Growth of Implanted Ehrlich’s Ascites Tumor in Mice
Source: PLoS One. 2015 Jul 2;10(7):e0132089. doi: 10.1371/journal.pone.0132089 (PMC4489743; doi:10.1371/journal.pone.0132089)
Supplement: S1 Table — Blood hematology was carried out after 3 months of 2-DG feeding before tumor implantation. (DOC) [file pone.0132089.s002.doc]

| **Parameters** | **Control** | **0.2% 2-DG** | **0.4% 2-DG** |
| --- | --- | --- | --- |
| RBC (10^6^ cells µl¯^1^) | 8.76±0.23 | 8.28±0.11 | 7.81±0.26 |
| Haemoglobin (gdl¯^1^) | 13.03±0.24 | 12.1±0.18 | 12.07±0.37 |
| Total leukocytes (10^3^ cells µl¯^1^ ) | 5.7±0.74 | 7.5±0.63* | 6.3±0.23 |
| Platelets (10^3^ cells µl¯^1^) | 692.0±29.1 | 673.7±52.3 | 672.8±47.6 |
| Lymphocytes (10^3^ cells µl¯^1^) | 2.8±0.5 | 3.7±0.28 | 2.9±0.49 |
| Granulocytes (10^3^ cells µl¯^1^) | 2.9±0.49 | 4.018±0.45 | 2.66±0.26 |

Note**:** Values are means ±SE from n = 4 - 6 mice/group and * indicates significance at p < 0.05.
